# Supplementary material for: Cytotoxic Polyketides from the Marine Sponge-Derived Fungus Pestalotiopsis heterocornis XWS03F09
Source: Molecules. 2019 Jul 22;24(14):2655. doi: 10.3390/molecules24142655 (PMC6680542; doi:10.3390/molecules24142655)
Supplement: Supplementary file 1 [file molecules-24-02655-s001.pdf]

Supporting Information

Cytotoxic polyketides from the marine sponge-derived fungus *Pestalotiopsis heterocornis* XWS03F09

Hui Lei[a,1], Jing Lei[a,1], Xuefeng Zhou[b], Mei Hu[a], Hong Niu[a], Can Song[a], Shiwei Chen[a], Yonghong Liu[b,*], Dan Zhang[a,*]

[a] School of Pharmacy, Southwest Medical University, Luzhou, Sichuan 646000, PR China;

[b] CAS Key Laboratory of Tropical Marine Bio-resources and Ecology, Guangdong Key Laboratory of Marine Materia Medica, RNAM Center for Marine

Microbiology, South China Sea Institute of Oceanology, Chinese Academy of Sciences, Guangzhou 510301, P. R. China

* Corresponding authors at: School of Pharmacy, Southwest Medical University, Luzhou, Sichuan 646000, PR China

** Corresponding authors.

E-mail addresses: yonghongliu@scsio.ac.cn (Y. Liu); zhangdan@swmu.edu.cn (D. Zhang)

[1] These authors contributed equally to this work.

Figure S1. HRESI-MS spectrum of the new compound **1**

Figure S2. ^{1}H NMR (600 MHz, CD_3OD) spectrum of the new compound **1**

Figure S3. ^{13}C NMR (150 MHz, CD_3OD) spectrum of the new compound **1**

Figure S4. HSQC spectrum of the new compound **1**

Figure S5. HMBC spectrum of the new compound **1**

Figure S6. COSY spectrum of the new compound **1**

Figure S7. NOESY spectrum of the new compound **1**

Figure S8. HRESI-MS spectrum of the new compound **2**

Figure S9. ^{1}H NMR (600 MHz, CD_3OD) spectrum of the new compound **2**

Figure S10. ^{13}C NMR (150 MHz, CD_3OD) spectrum of the new compound **2**

Figure S11. HSQC spectrum of the new compound **2**

Figure S12. HMBC spectrum of the new compound **2**

Figure S13. COSY spectrum of the new compound **2**

Figure S14. NOESY spectrum of the new compound **2**

Figure S15. HRESI-MS spectrum of the new compound **3/4**

Figure S16. ^{1}H NMR (600 MHz, CD_3OD) spectrum of the new compound **3/4**

Figure S17. ^{13}C NMR (150 MHz, CD_3OD) spectrum of the new compound **3/4**

Figure S18. HSQC spectrum of the new compound **3/4**

Figure S19. HMBC spectrum of the new compound **3/4**

Figure S20. COSY spectrum of the new compound **3/4**

Figure S21. NOESY spectrum of the new compound **3/4**

Mass Spectrum SmartFormula Report

Analysis Info

Analysis Name	D:\Data\MS\data\201806\yangbin_L9_pos_31_01_5004.d	Acquisition Date	6/25/2018 10:44:40 AM
Method	LC_Direct Infusion_pos_100-1000mz.m	Operator	SCSIO
Sample Name	yangbin_L9_pos	Instrument	maXis 255552.00029
Comment			

Acquisition Parameter

Source Type	ESI	Ion Polarity	Positive	Set Nebulizer	0.4 Bar
Focus	Active	Set Capillary	4500 V	Set Dry Heater	180 °C
Scan Begin	100 m/z	Set End Plate Offset	-500 V	Set Dry Gas	4.0 l/min
Scan End	2000 m/z	Set Charging Voltage	0 V	Set Divert Valve	Waste
		Set Corona	0 nA	Set APCI Heater	0 °C

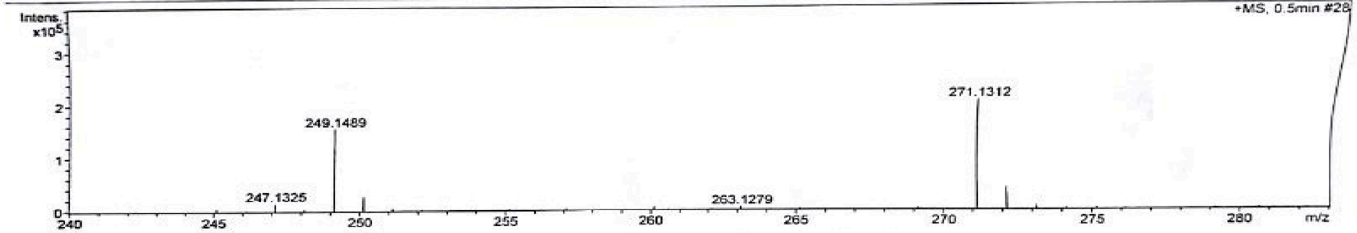

Meas. m/z	#	Ion Formula	Score	m/z	err [ppm]	err [mDa]	mSigma	rdb	e⁻ Conf	N-Rule
249.1489	1	C15H21O3	100.00	249.1485	1.4	0.4	3.9	5.5	even	ok
271.1312	1	C15H20NaO3	100.00	271.1305	-2.8	-0.8	20.2	5.5	even	ok
497.2886	1	C30H41O6	100.00	497.2898	2.3	1.1	21.6	10.5	even	ok
519.2712	1	C30H40NaO6	100.00	519.2717	0.9	0.5	26.7	10.5	even	ok

yangbin_L9_pos_31_01_5004.d
Bruker Compass DataAnalysis 4.1 printed: 6/25/2018 10:50:03 AM by: SCSIO Page 1 of 1

Figure 1. HRESI-MS spectrum of the new compound **1.**

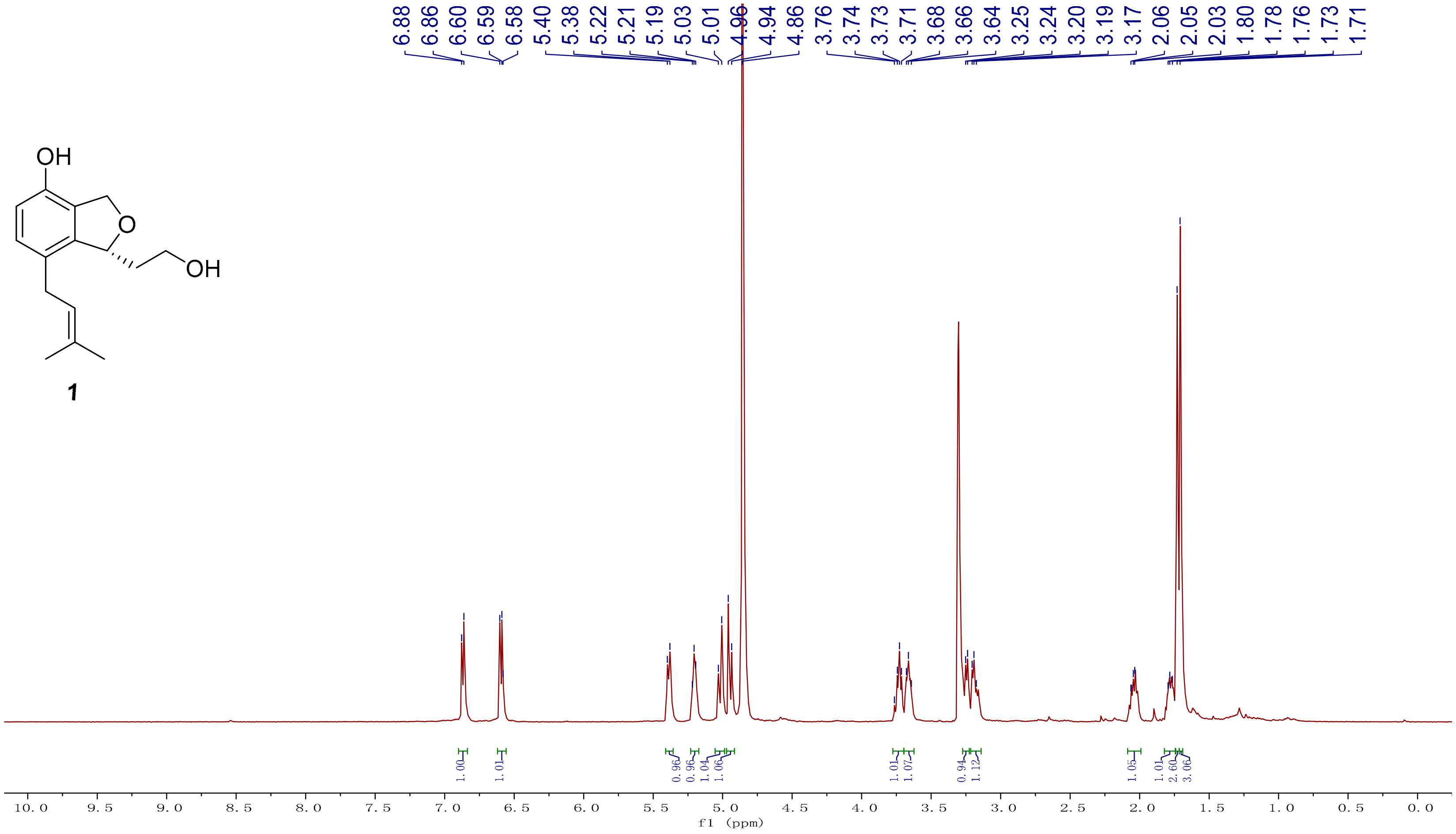

Figure S2. ^{1}H NMR (600 MHz, CD_3OD) spectrum of the new compound **1**

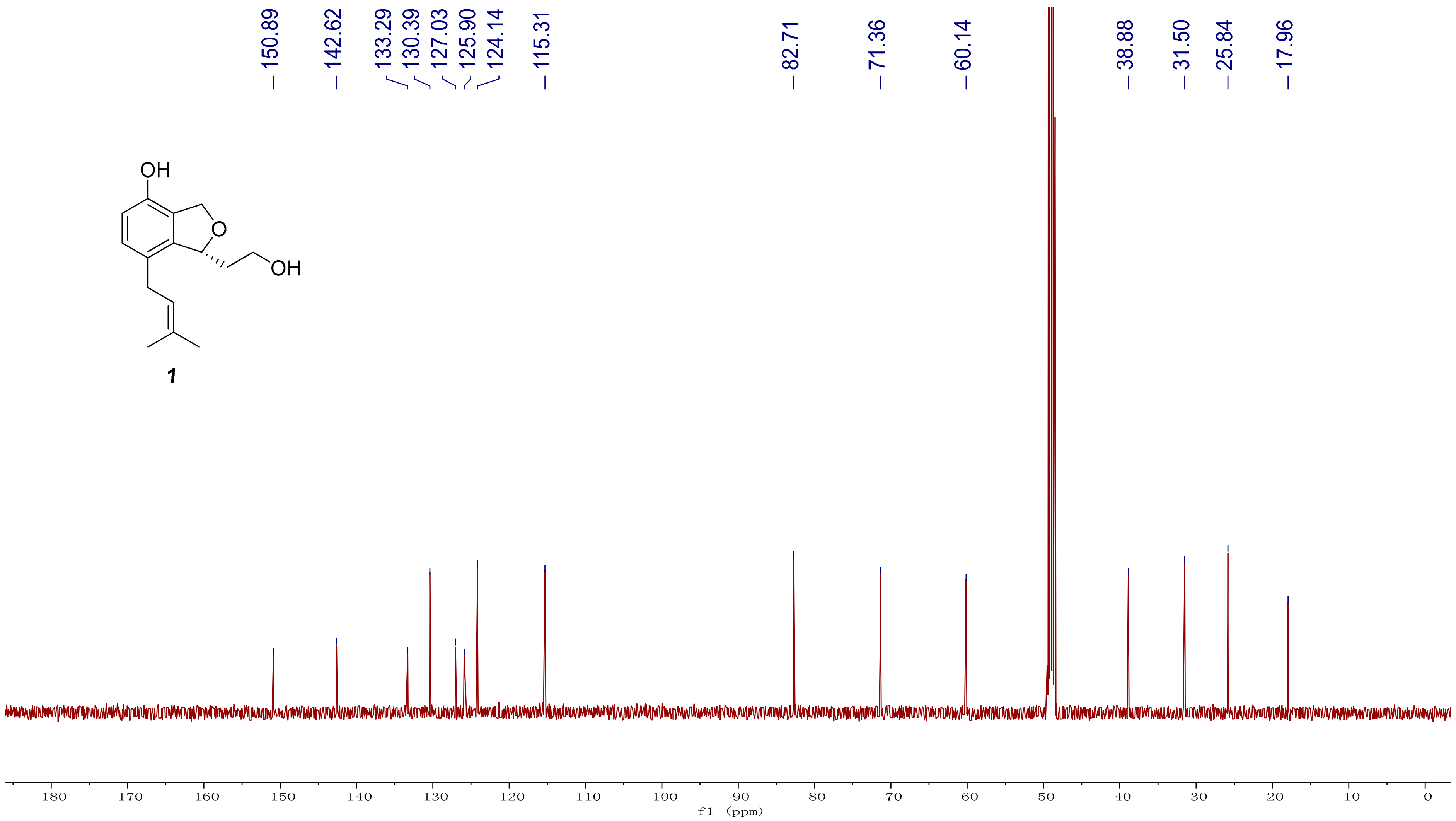

Figure S3. ^{13}C NMR (150 MHz, CD_3OD) spectrum of the new compound **1**

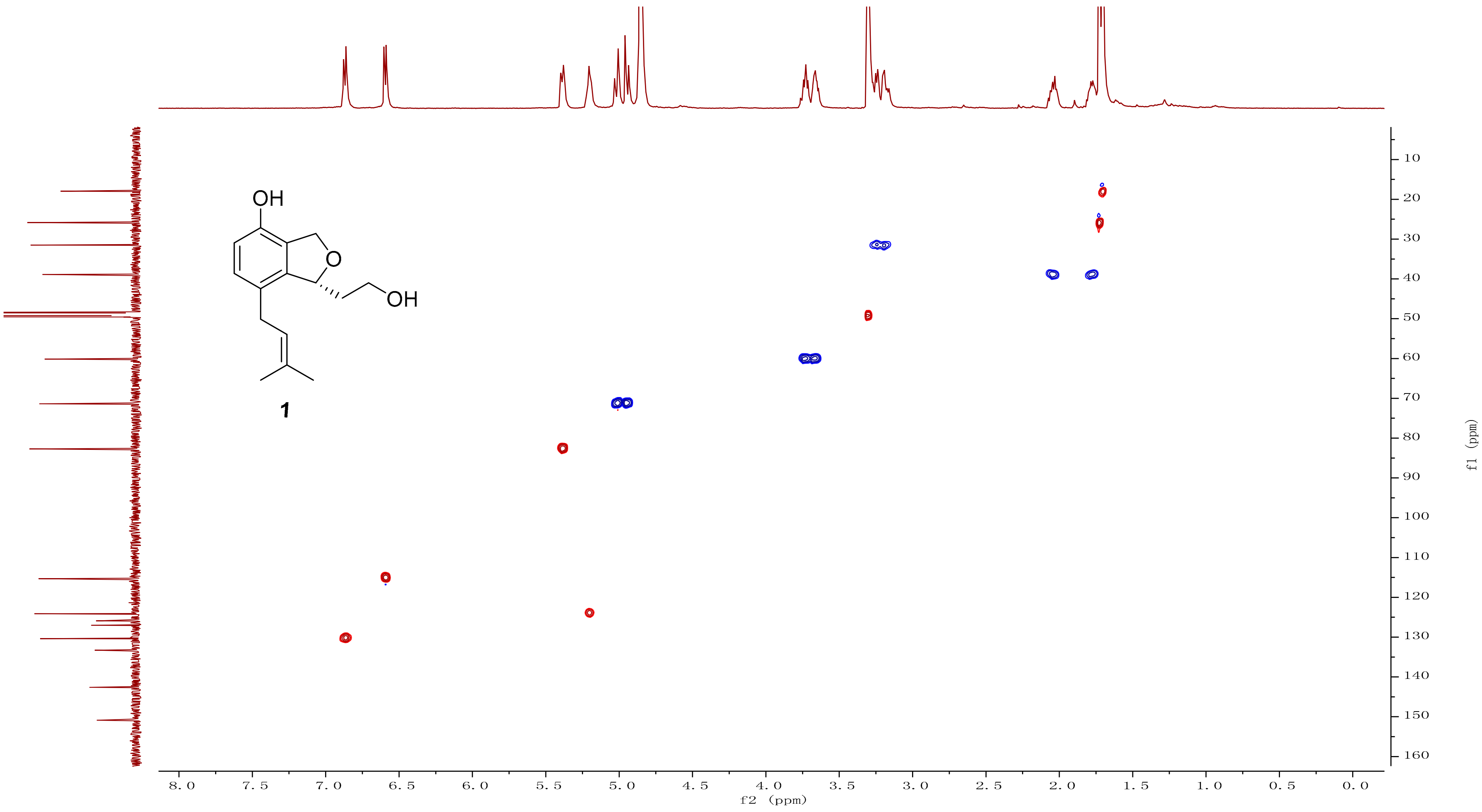

Figure S4. HSQC spectrum of the new compound **1**

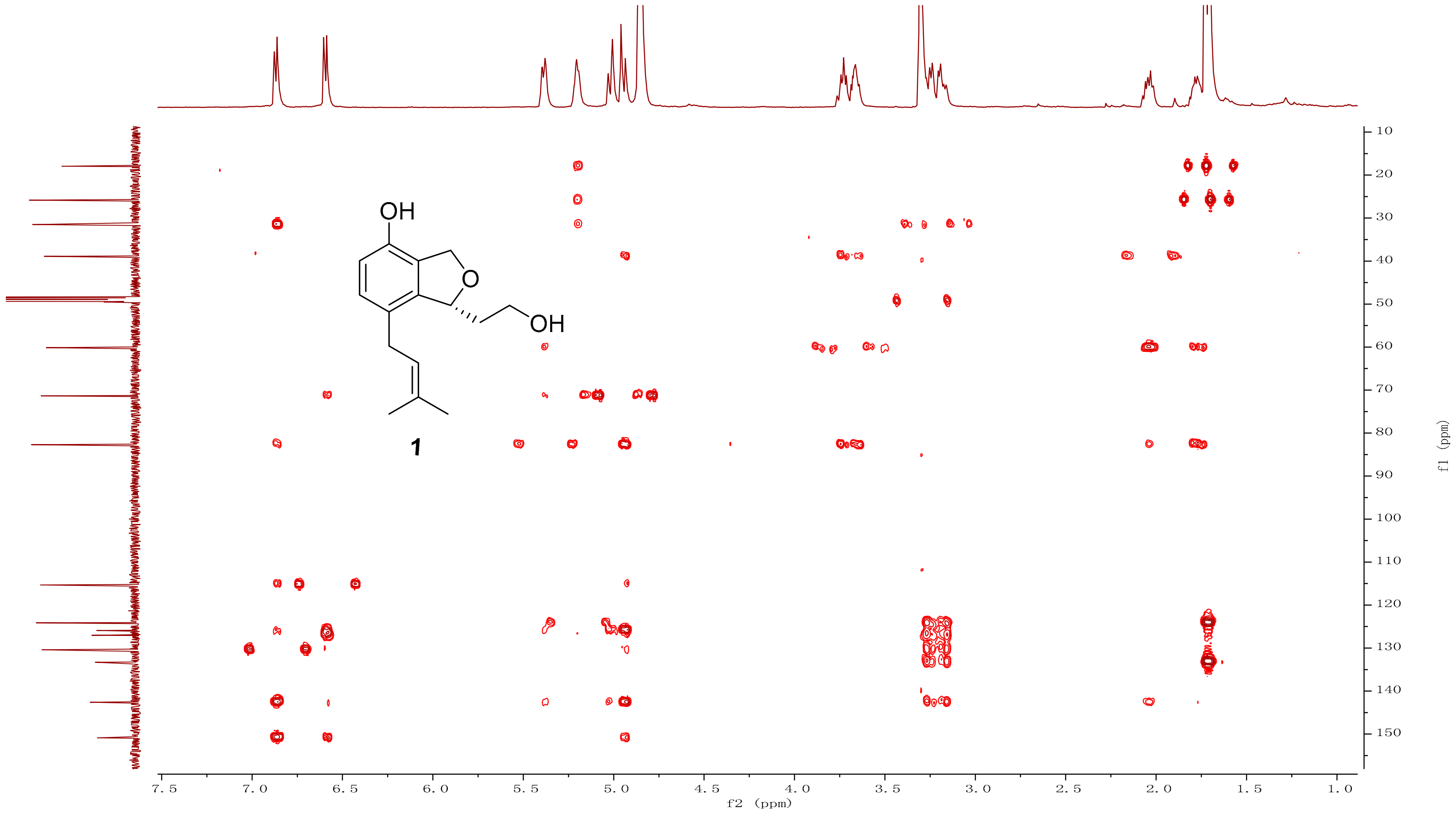

Figure S5. HMBC spectrum of the new compound **1**

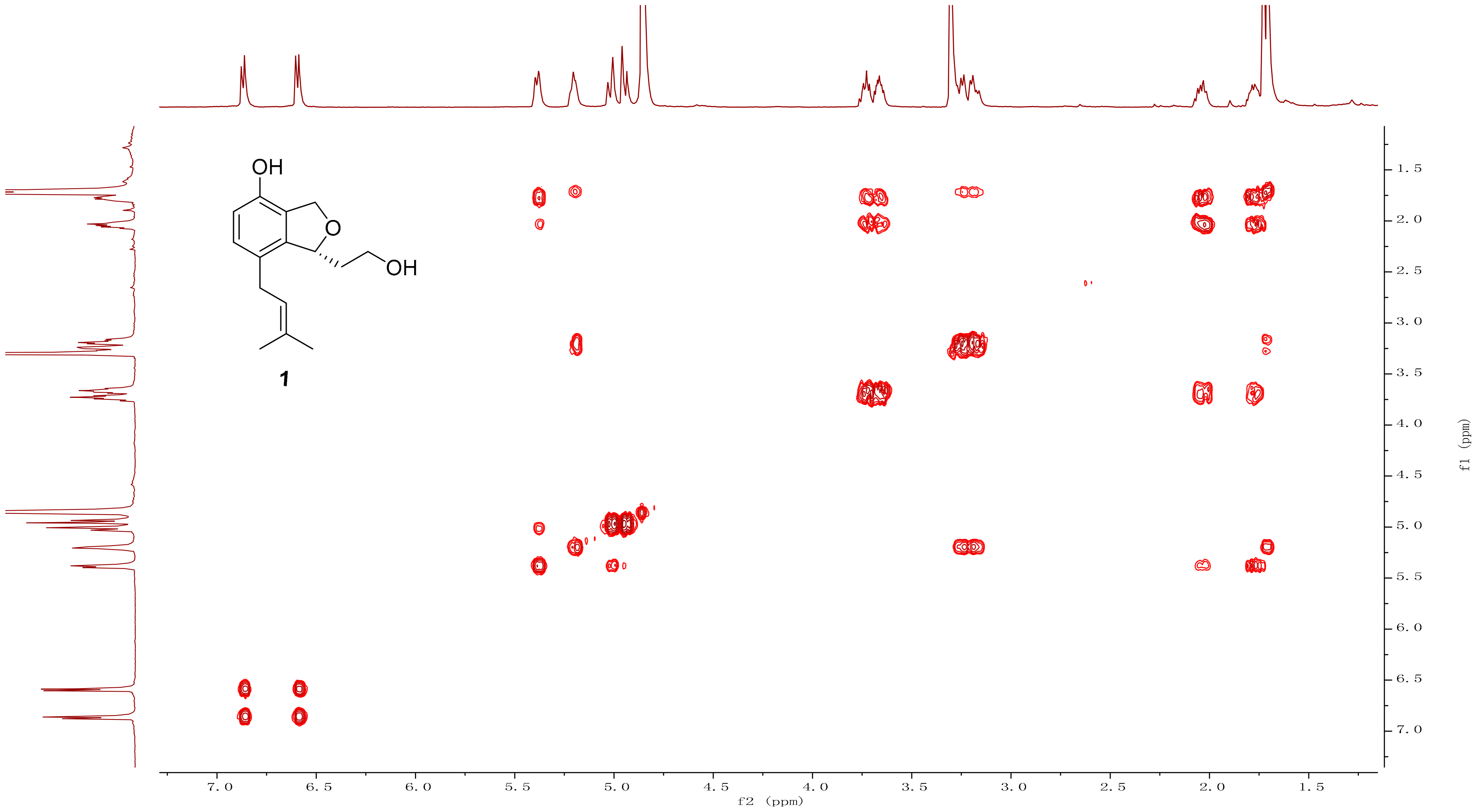

Figure S6. COSY spectrum of the new compound **1**

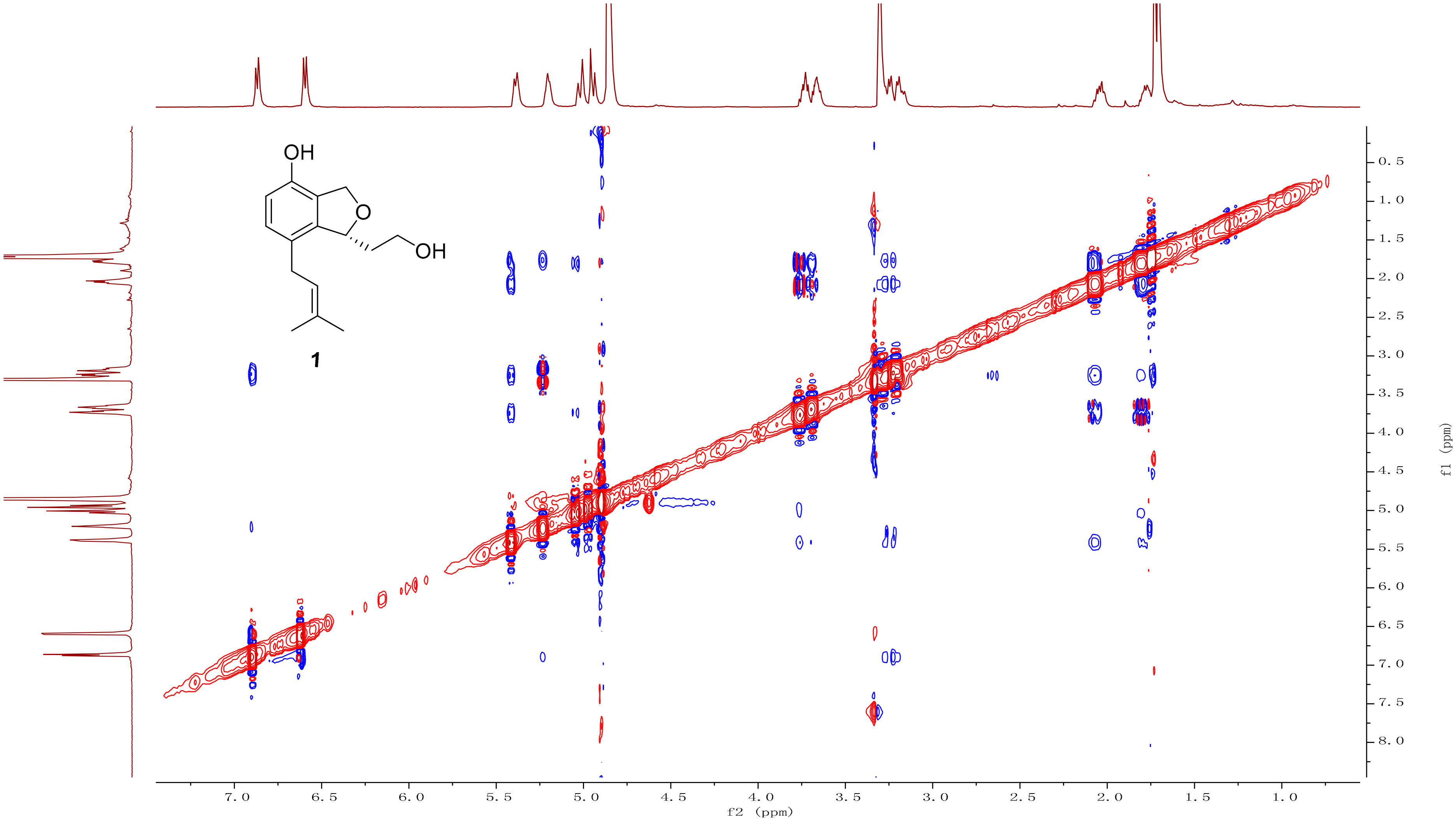

Figure S7. NOESY spectrum of the new compound **1**

Mass Spectrum SmartFormula Report

Analysis Info

Analysis Name	D:\Data\MS\data\201806\yangbin_L15_pos_32_01_5005.d	Acquisition Date	6/25/2018 10:48:06 AM
Method	LC_Direct Infusion_pos_100-1000mz.m	Operator	SCSIO
Sample Name	yangbin_L15_pos	Instrument	maXis 255552.00029
Comment			

Acquisition Parameter

Source Type	ESI	Ion Polarity	Positive	Set Nebulizer	0.4 Bar
Focus	Active	Set Capillary	4500 V	Set Dry Heater	180 °C
Scan Begin	100 m/z	Set End Plate Offset	-500 V	Set Dry Gas	4.0 l/min
Scan End	2000 m/z	Set Charging Voltage	0 V	Set Divert Valve	Waste
		Set Corona	0 nA	Set APCI Heater	0 °C

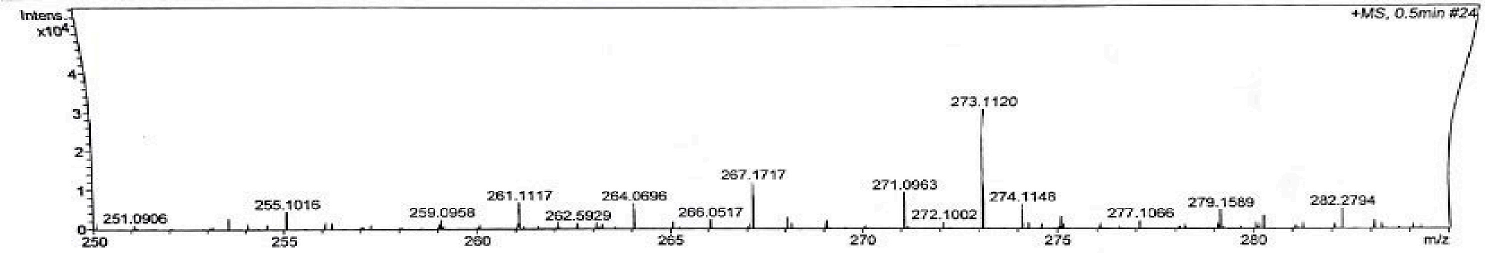

Meas. m/z	#	Ion Formula	Score	m/z	err [ppm]	err [mDa]	mSigma	rdb	e⁻ Conf	N-Rule
273.1120	1	C16H17O4	100.00	273.1121	-0.4	-0.1	20.5	8.5	even	ok

yangbin_L15_pos_32_01_5005.d
Bruker Compass DataAnalysis 4.1 printed: 6/25/2018 10:52:36 AM by: SCSIO Page 1 of 1

Figure S8. HRESI-MS spectrum of the new compound **2**

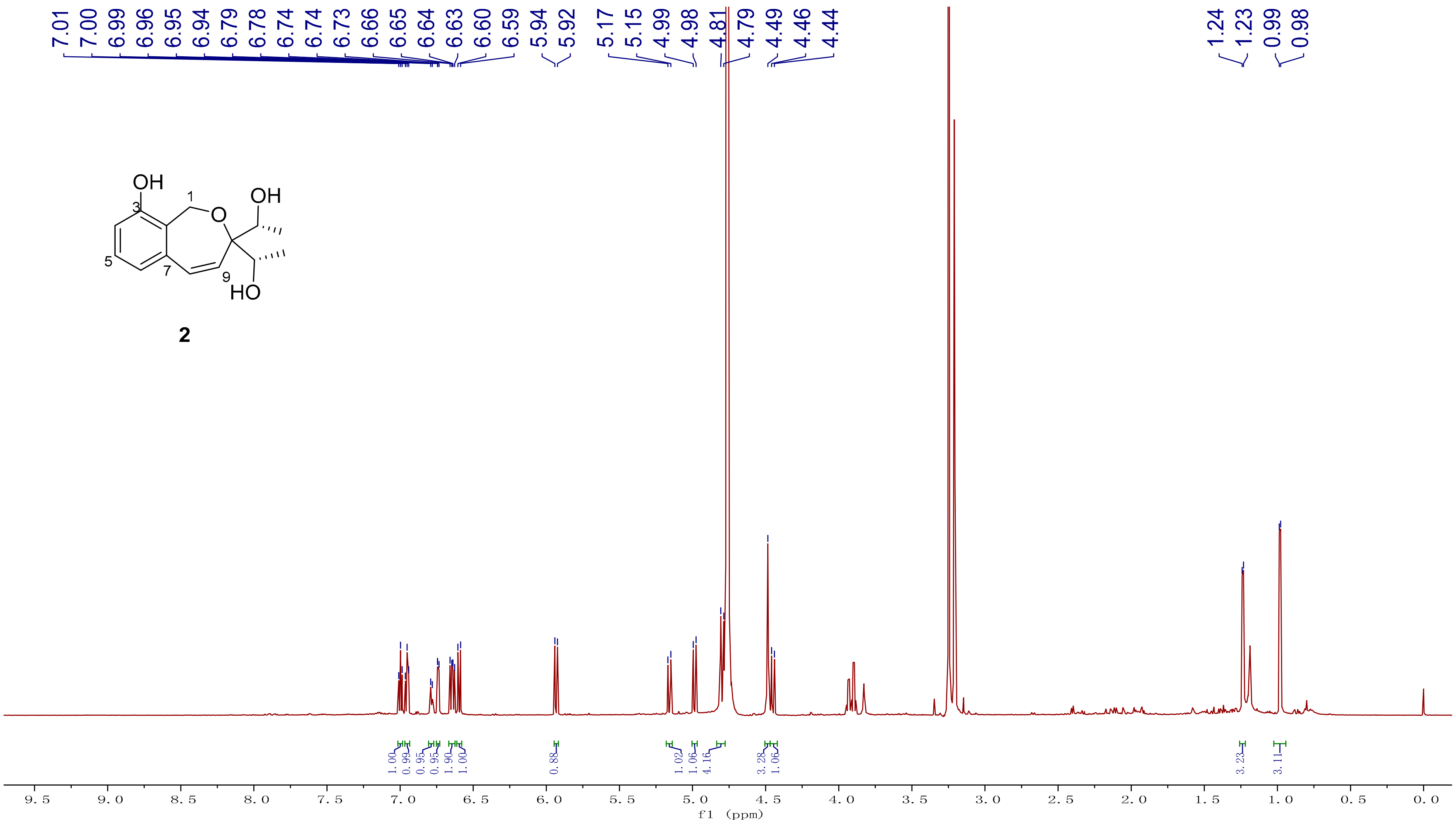

Figure S9. ^{1}H NMR (600 MHz, CD_3OD) spectrum of the new compound **2**

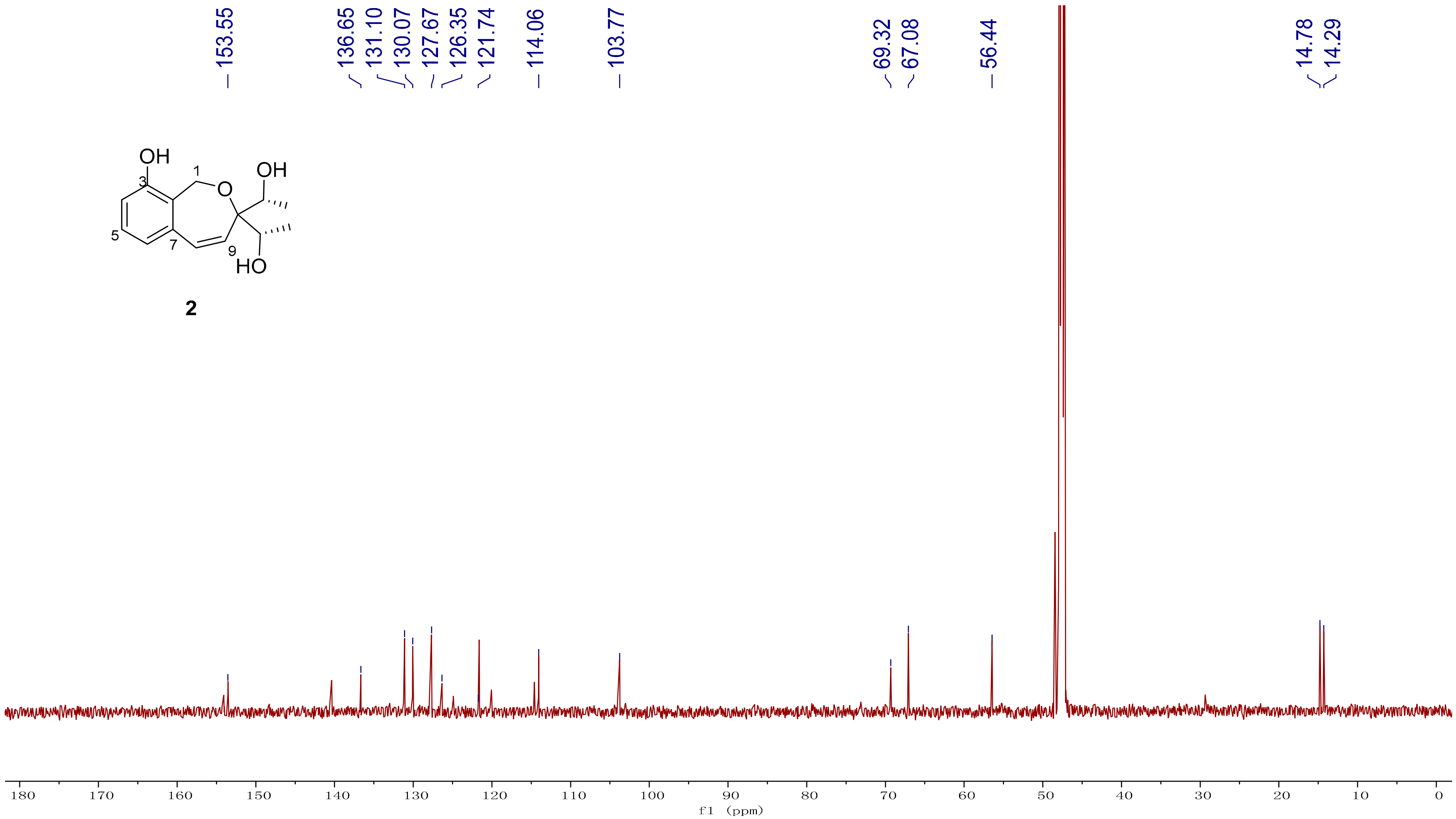

Figure S10. ^{13}C NMR (150 MHz, CD_3OD) spectrum of the newcompound **2**

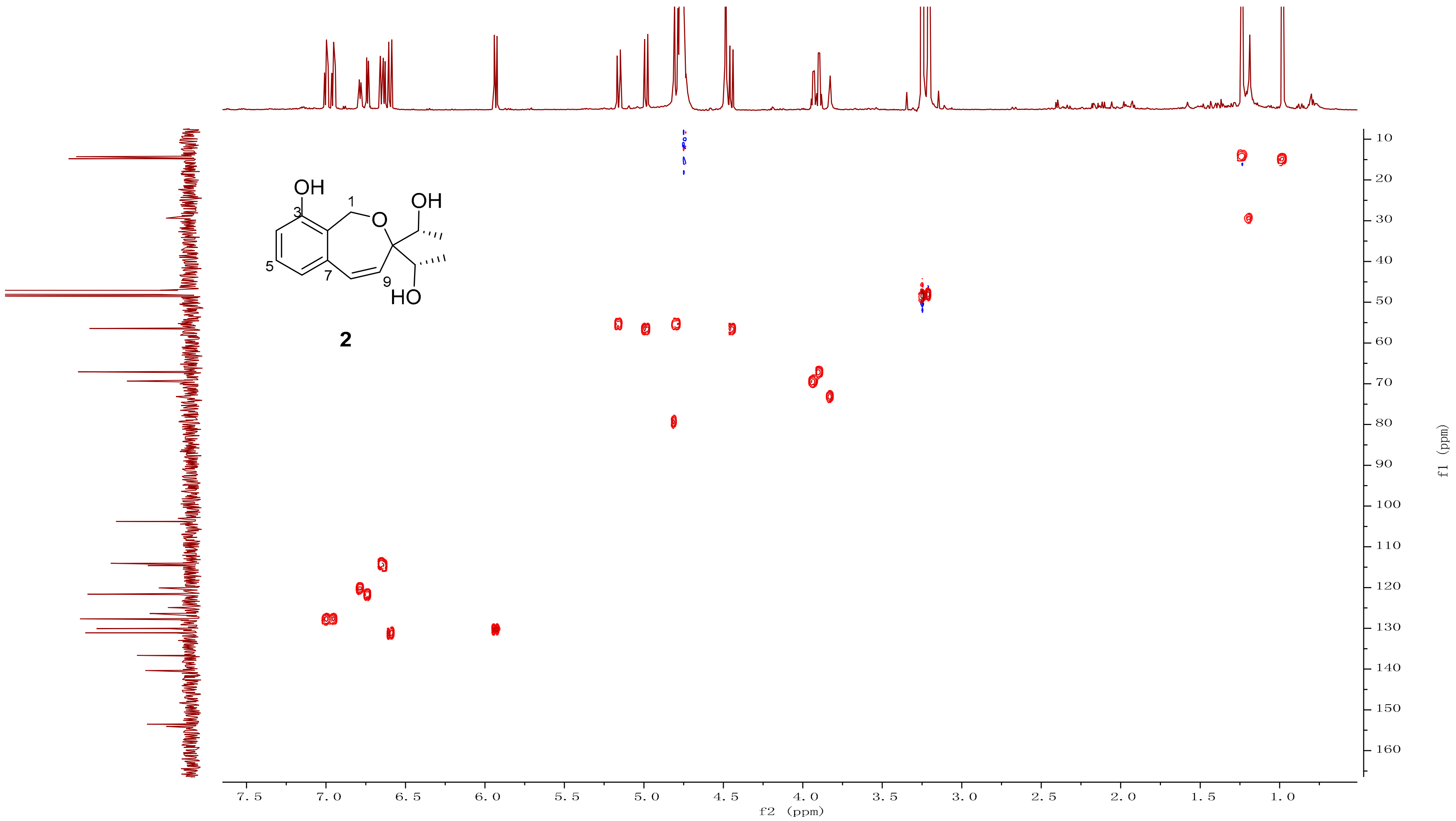

Figure S11. HSQC spectrum of the new compound **2**

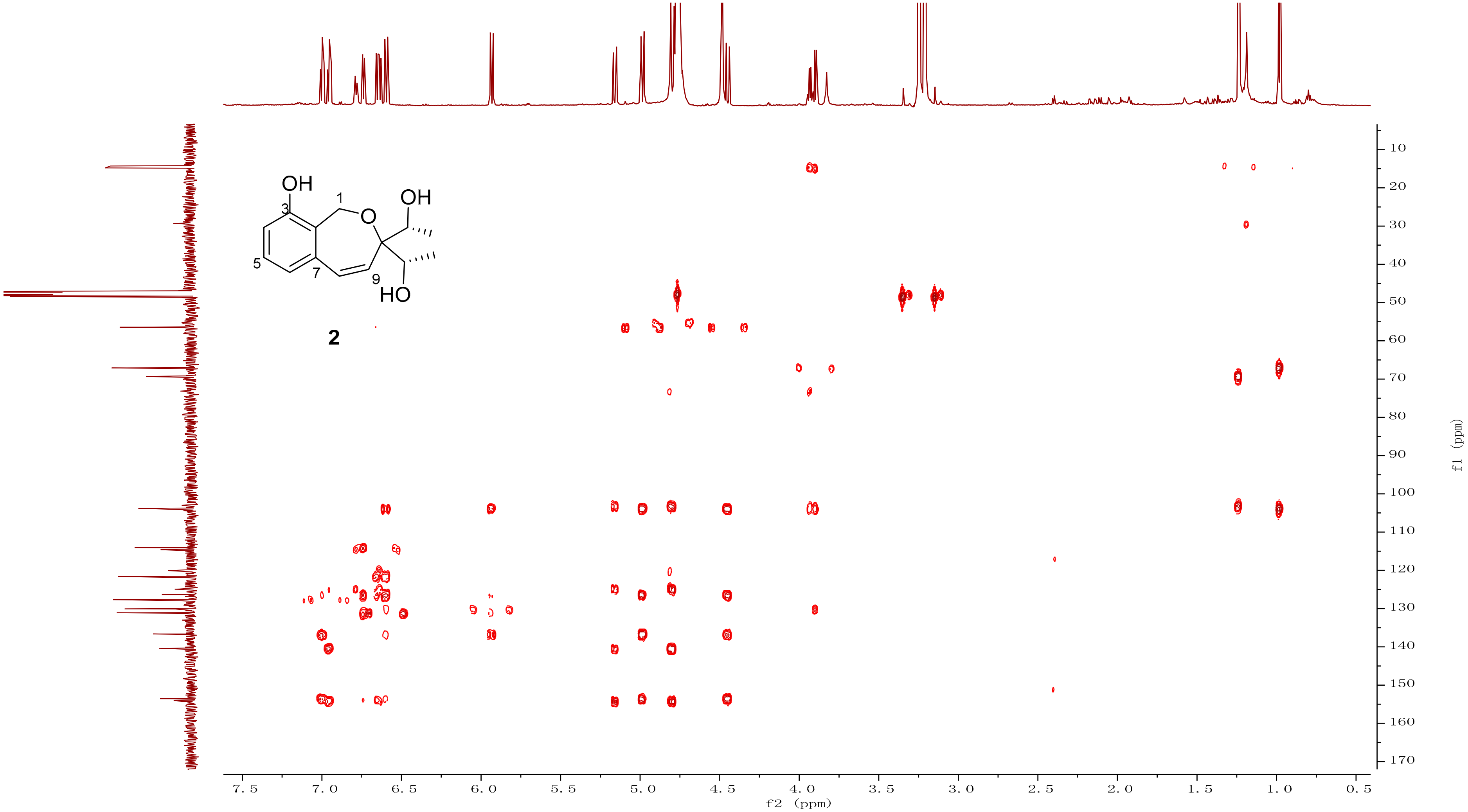

Figure S12. HMBC spectrum of the new compound **2**

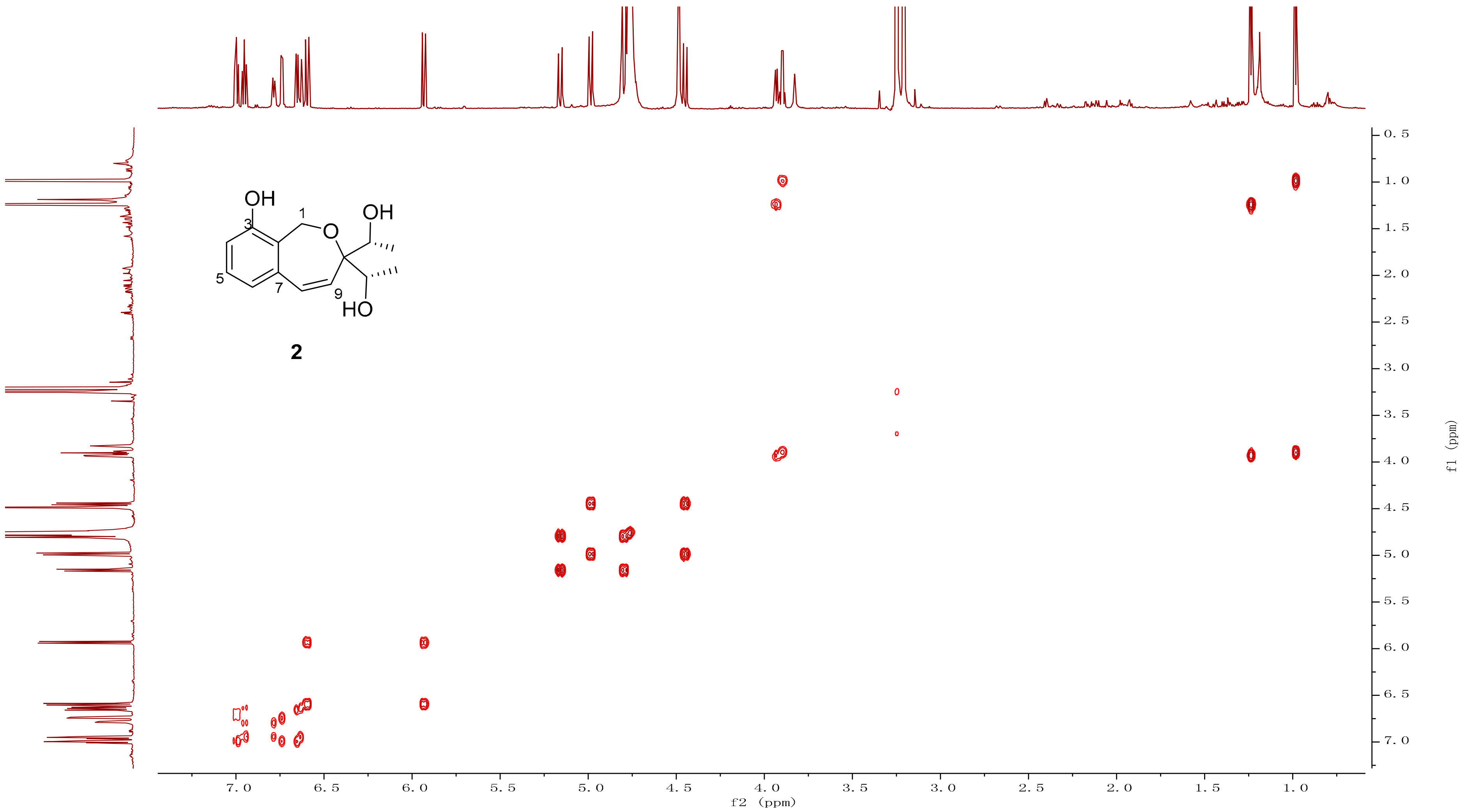

Figure S13. COSY spectrum of the new compound **2**

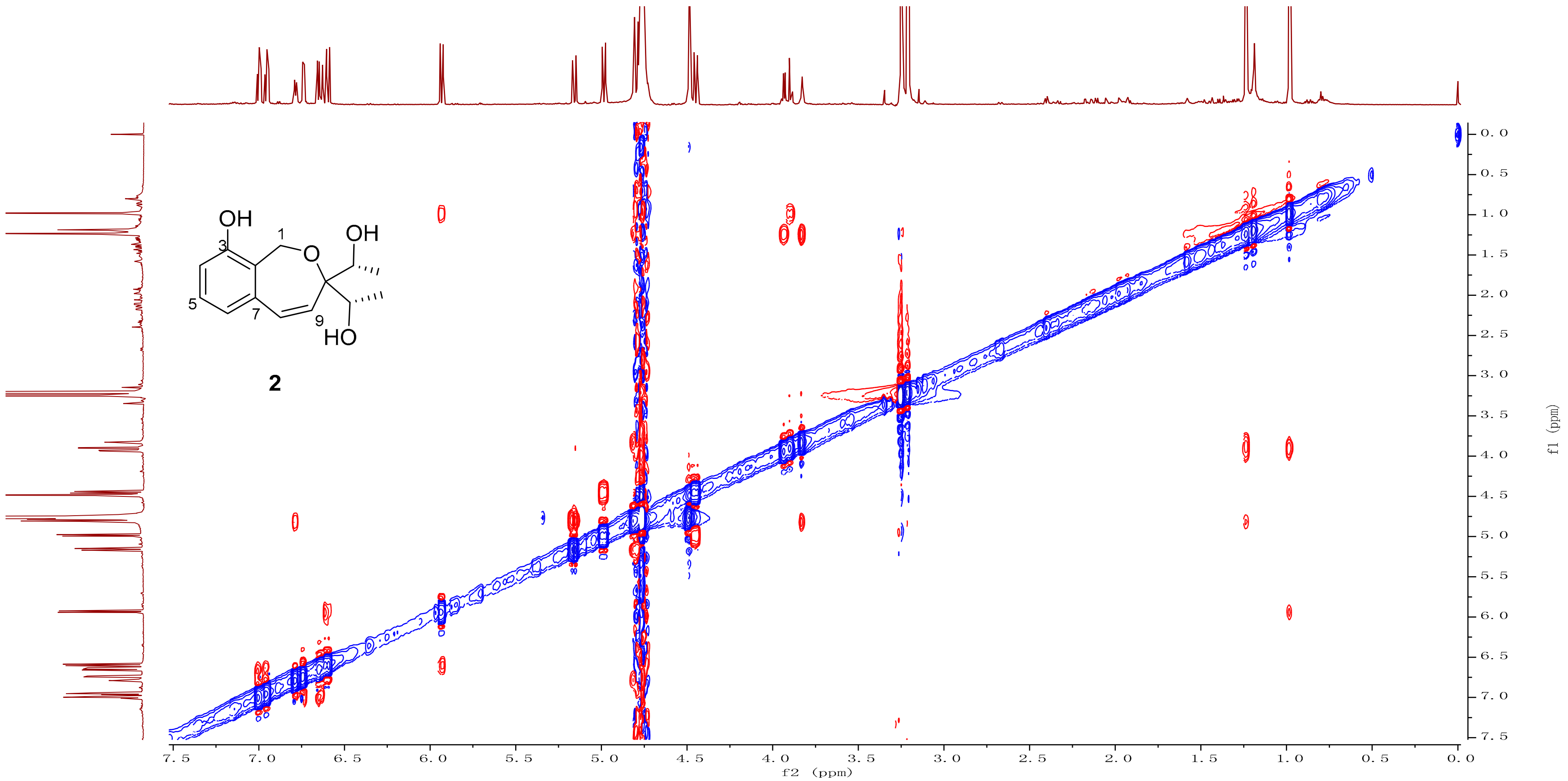

Figure S14. NOESY spectrum of the new compound **2**

Mass Spectrum Molecular Formula Report

Analysis Info

Analysis Name	D:\Data\20151216CEYANG\new folder\NEU-19_1-C,1_01_6265.d	Acquisition Date	12/16/2015 3:33:42 PM
Method	20131026_ceyang.m	Instrument / Ser#	Bruker Customer
Sample Name	NEU-19	Operator	micrOTOF-Q 125
Comment			

Acquisition Parameter

Source Type	ESI	Ion Polarity	Positive	Set Nebulizer	1.2 Bar
Focus	Active	Set Capillary	4500 V	Set Dry Heater	180 °C
Scan Begin	50 m/z	Set End Plate Offset	-500 V	Set Dry Gas	8.0 l/min
Scan End	3000 m/z	Set Collision Cell RF	100.0 Vpp	Set Divert Valve	Source

Generate Molecular Formula Parameter

Formula, min.		
Formula, max.		
Measured m/z	Tolerance	Charge
Check Valence	Minimum	Maximum
Nirogen Rule	Electron Configuration	
Filter H/C Ratio	Minimum	Maximum
Estimate Carbon		

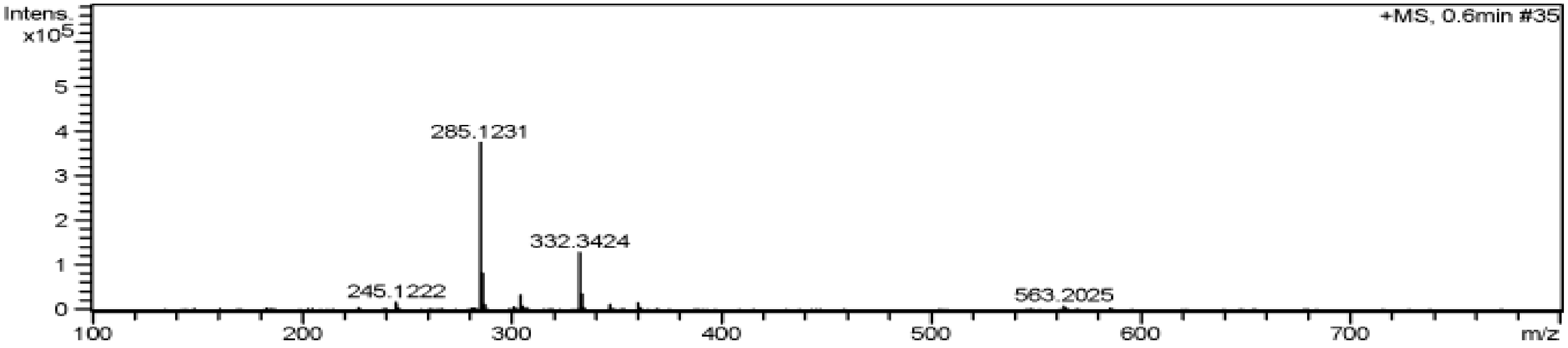

Figure S15. HRESI-MS spectrum of the new compound **3/4**

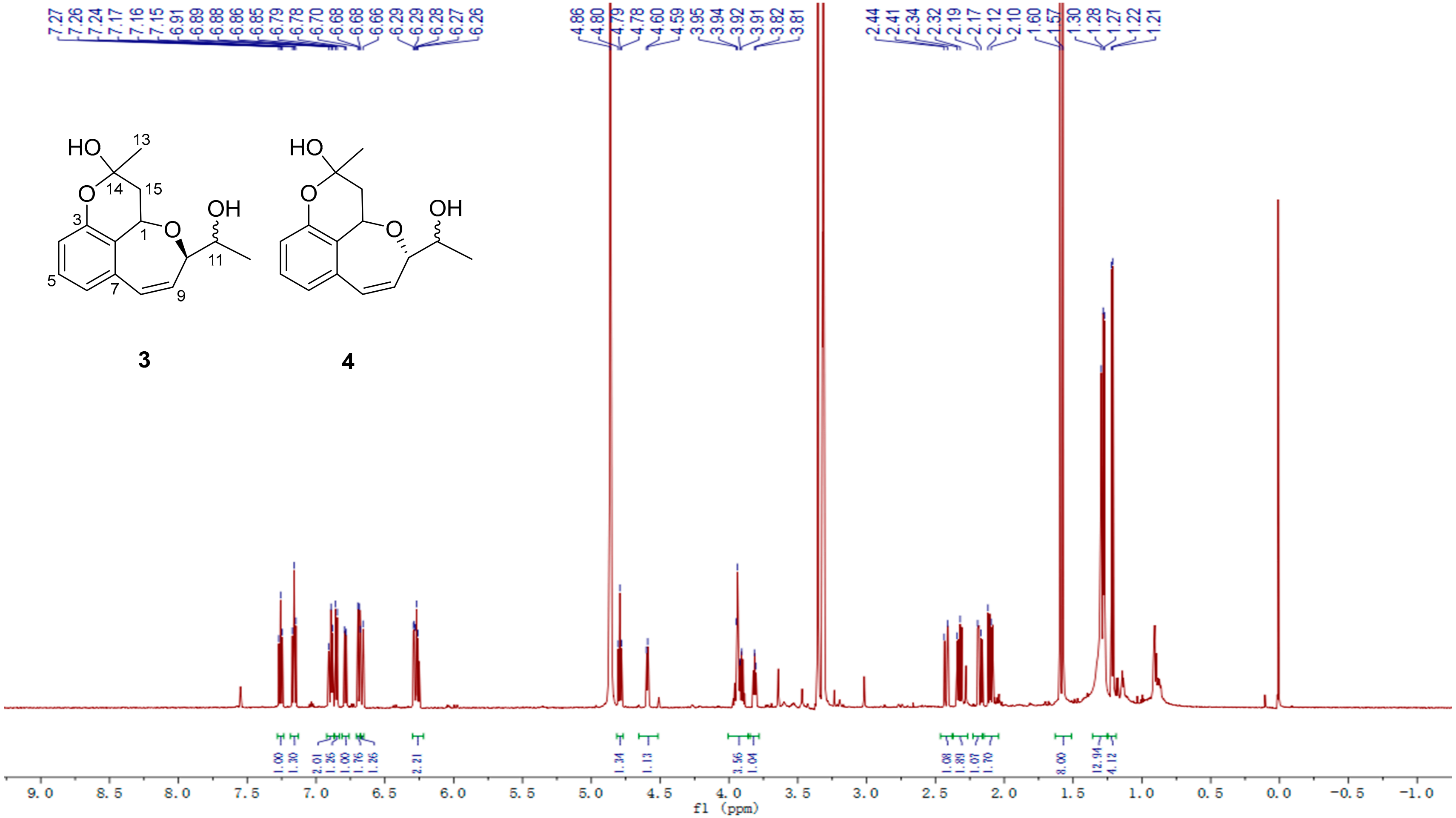

Figure S16. ^{1}H NMR (600 MHz, CD_3OD) spectrum of the new compound **3/4**

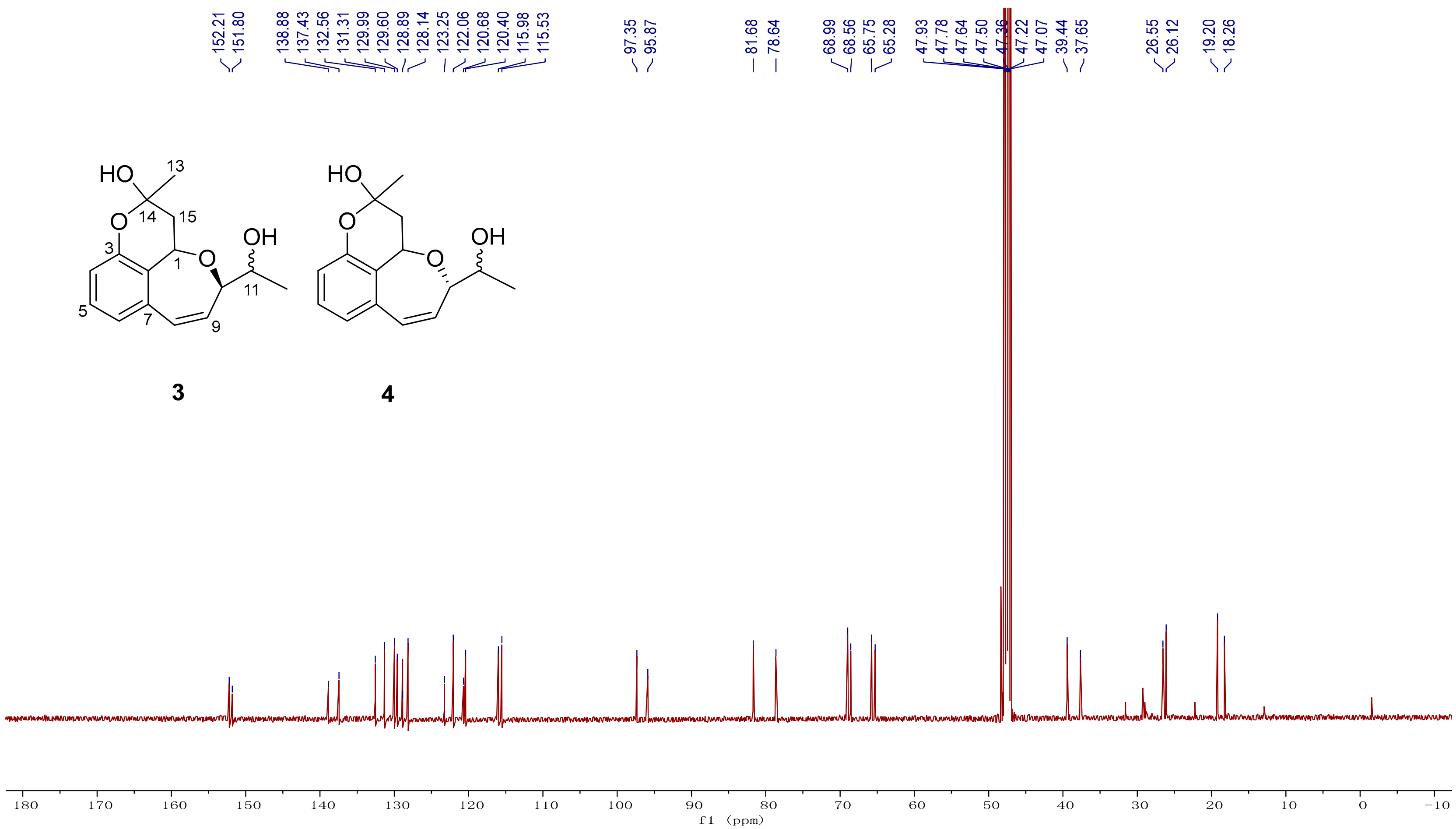

Fiure S17. ^{13}C NMR (150 MHz, CD_3OD) spectrum of the new compound **3**/**4**

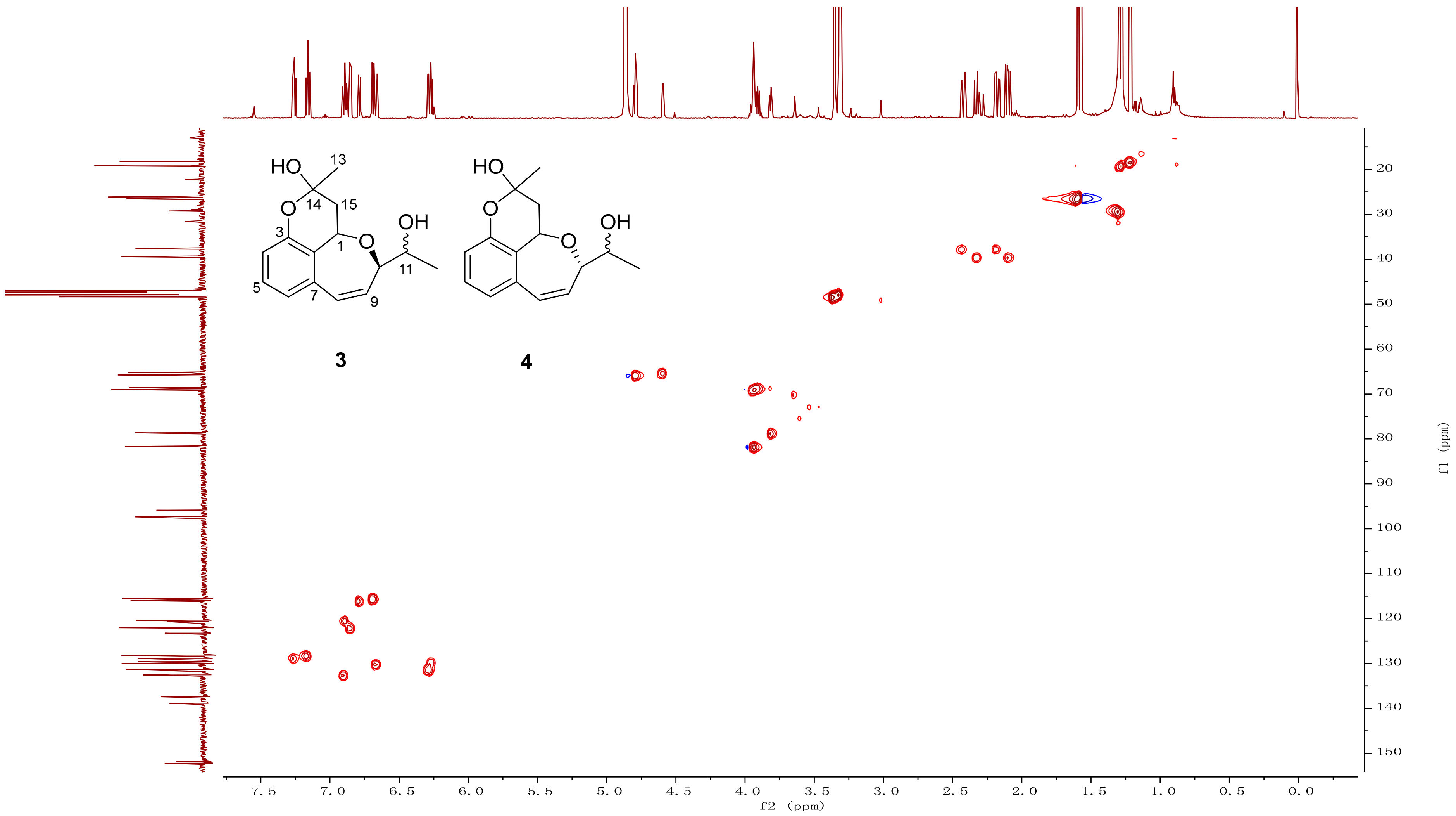

Figure S18. HSQC spectrum of the new compound **3/4**

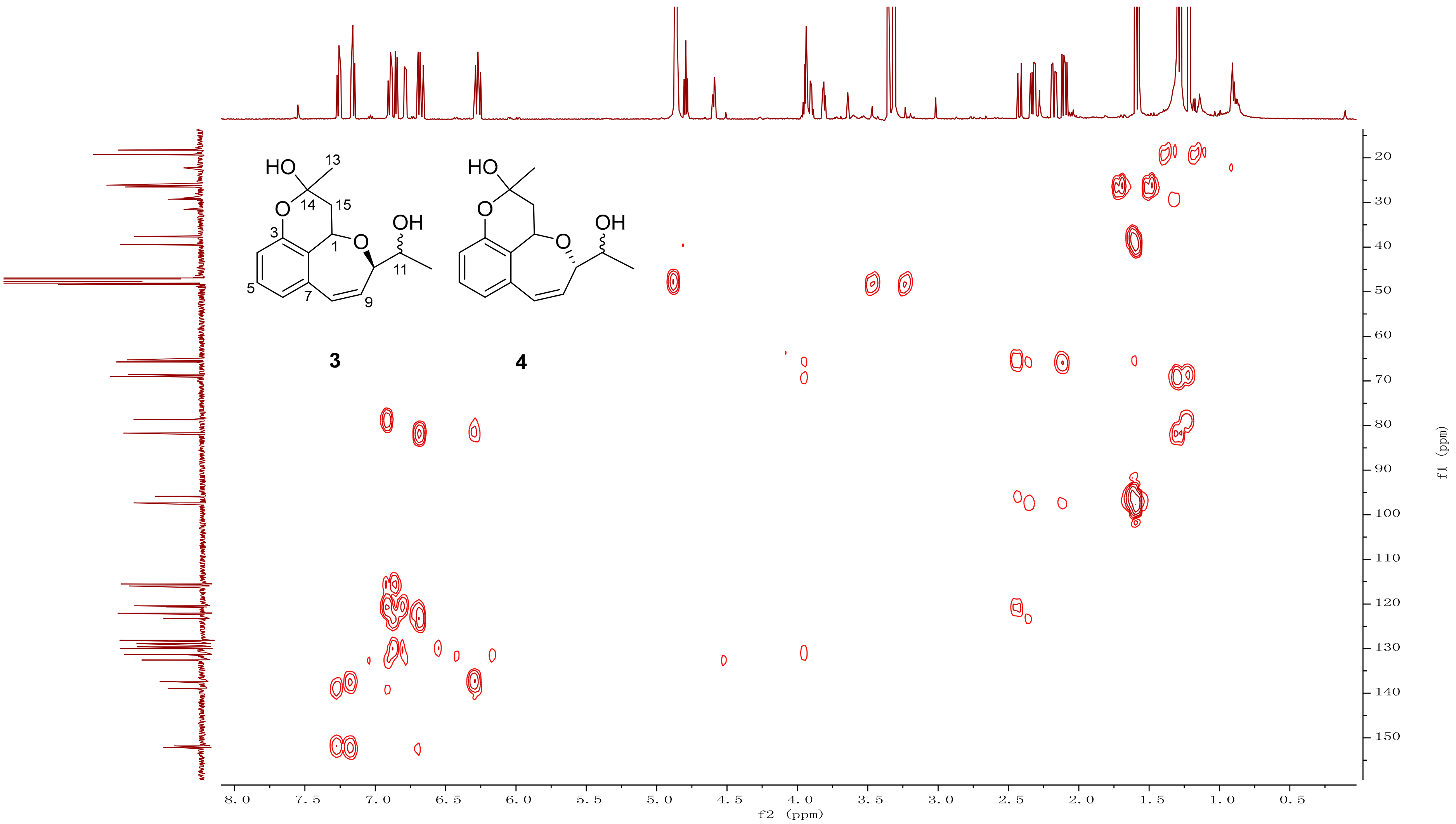

Figure S19. HMBC spectrum of the new compound **3/4**

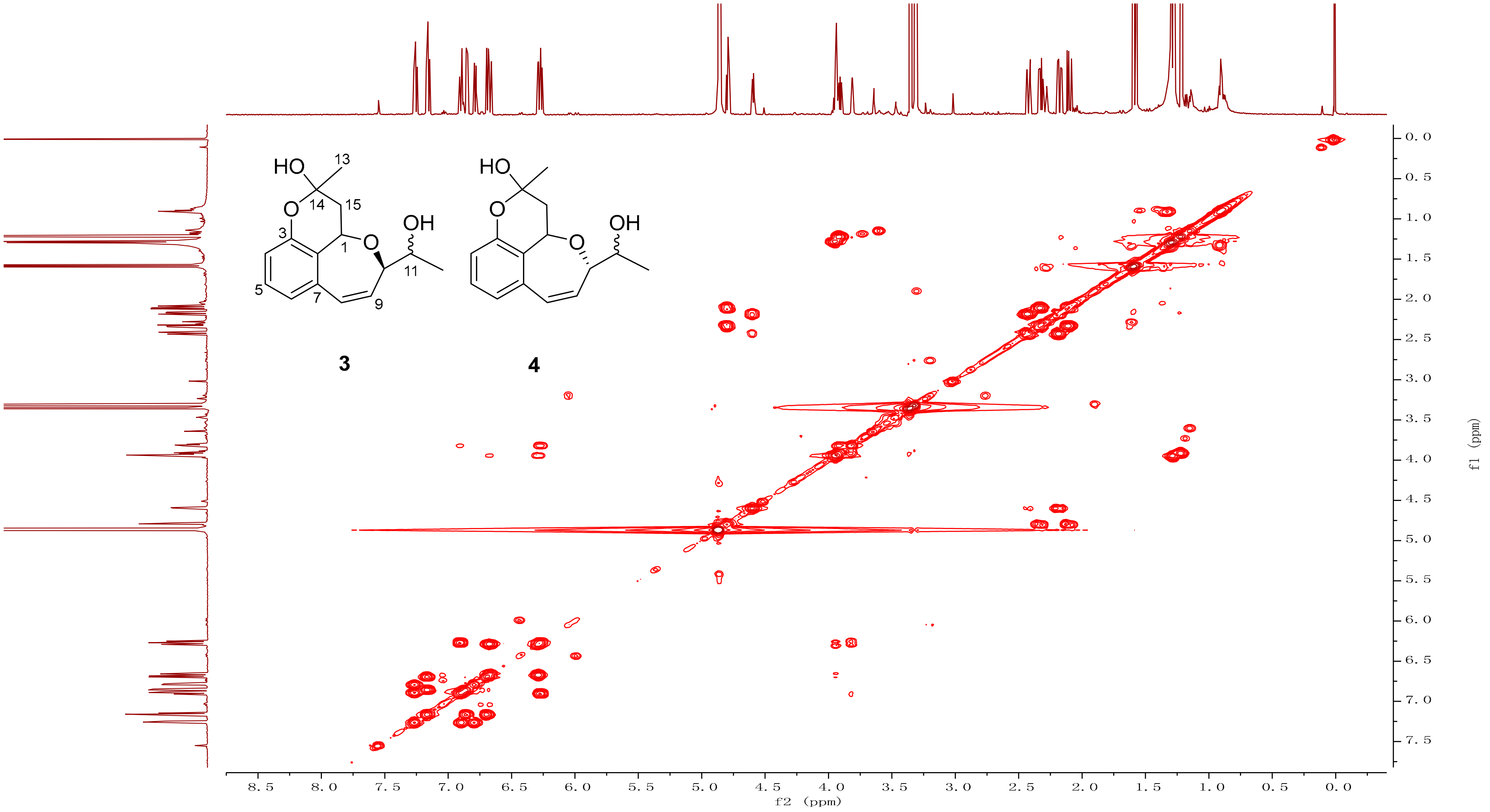

Figure S20. COSY spectrum of the new compound **3/4**

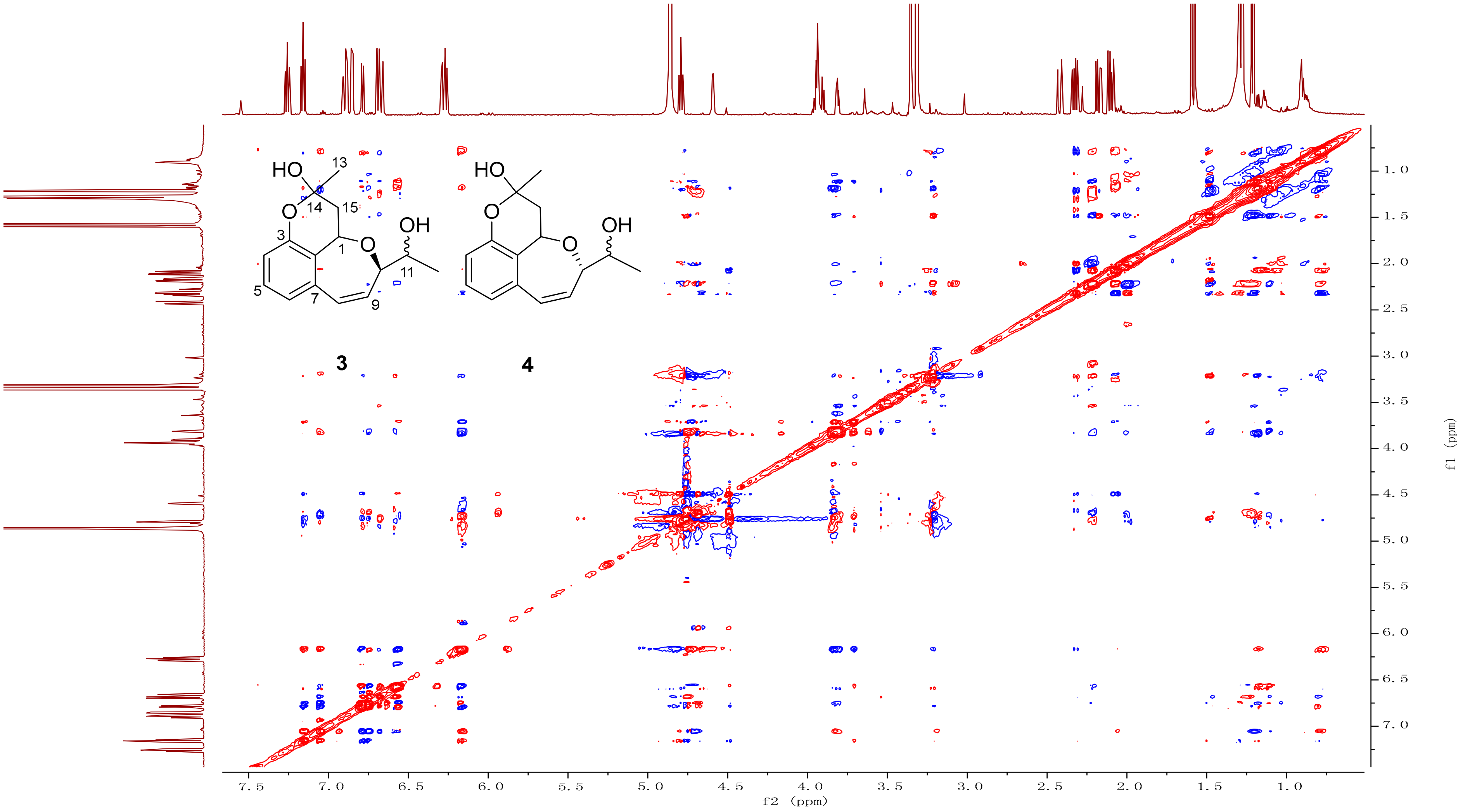

Figure S21. NOESY spectrum of the new compound **3/4**
